# Strengthening antimicrobial resistance governance in Europe: a coordinated one health approach

**DOI:** 10.1016/j.lanepe.2025.101540

**Published:** 2025-11-18

**Authors:** Benjamin Davido, Sofia Ny, Corine van Lingen, Christine Årdal, Laura Alonso Irujo, Sofia Linnros, Lucie Collineau, Nerea Gonzalez Hernandez, Marta Gutierrez Rubio, Giel van de Laar, Lida Politi, Brian Kristensen, Ana Álvarez-Fernández, Yohann Lacotte, Agathe Claude, Marie-Cécile Ploy, JAMRAI2 Executive Board Members, Laura Alonso Irujo, Ana Alvarez Fernandez, Fortunato D'Ancona, Victor Aníbal Lopez, Christine Ardal, Servane Bareille, Min Barnet, Anne Becker, Gitte Bekker, Michael Borg, Blanca Bradley Valdenegro, Els Broens, Ricardo Carapeto Garcia, Pieter-Jan Ceyssens, Agathe Claude, Lucie Collineau, Geoffrey Couraud, Christophe Dagot, Peter Damborg, Benjamin Davido, Julie Debouvere, Giulia Fadda, Rocío Fernández Urrusuno, Marie Fuentes-Braesch, Ane Fullaondo Zabala, Silvana Gastaldi, Jonathan Gómez Raja, Nerea Gonzalez Hernandez, Priscila Guerra, Marta Gutierrez, Evelyne de Graef, Annette Hulth, Katerina Iliopoulou, Claudia Isonne, Ulisa Jeyaratnam, Moira Kelly, Brian Kristensen, Asja Kunoe, Aapo Kuusipalo, Giel van de Laar, Ane Laburu Dañobeitia, Yohann Lacotte, Sarah Le Gall, Enric Limon Caceres, Corine van Lingen, Sofia Linnros, Maria del Pilar Lopez Acuña, Antonio Lopez, Maria Lopez Rodriguez, Luis Lucena Baeza, Roosmarijn Luiken, Jean-Yves Madec, Noémie Mercier, Signe Miang Jensen, Cristina Munoz, Ana Navarro Tamayo, María Núñez-Núñez, Sofia Ny, Minttu Palsola, Jose Ramon Paño, Amy Parrish, Marie-Cécile Ploy, Lida Politi, Miquel Pujol Rojo, Karina Rojas Salvador, Heike Schmitt, Uramaru Teinauri, Jose Luis Trillo Contreras, Richard Vaux, Dolores Verdoy Berastegi, Christophe Vermeulen, Maria J. Vilar, Isaura Wayop, Ute Wolff Sönksen, Maite Zabala Ugarteburu

**Affiliations:** a Maladies Infectieuses, Hôpital Raymond-Poincaré, Université Paris Saclay, AP-HP, 92380, Garches, France; b UMR1173, Université Versailles Saint-Quentin, 78180 Montigny-Le-Bretonneux, France; c Mission Ministérielle de la Prévention des infections et de l'antibiorésistance, Direction Générale de la Santé, 75007, Paris, France; d Public Health Agency of Sweden, Stockholm, Sweden; e Ministry of Health, Welfare and Sport, The Hague, Netherlands; f Norwegian Institute of Public Health, Oslo, Norway; g Fundación FCSAI, Spanish Agency of Medicines and Medical Devices, Madrid, Spain; h University of Lyon, French Agency for Food, Environmental and Occupational Health & Safety (ANSES), Lyon, France; i Biosistemak Institute for Health Systems Research, Bilbao, Spain; j National Public Health Organization, Athens, Greece; k Statens Serum Institut, Copenhagen, Denmark; l VINCat Program, Servei Català de la Salut-Departament de Salut, Catalonia, Spain; m Institut Català d'Oncologia, 08908, l’Hospitalet de Llobregat, Barcelona, Spain; n Université de Limoges, Inserm U1092 CHU Limoges, RESINFIT, Limoges, France

**Keywords:** AMR, Burden of disease, Antibiotic resistance, Europe

## Abstract

Antimicrobial resistance (AMR) causes over 35,000 deaths annually in the EU/EEA and is projected to result in 1.91 million global deaths each year by 2050. The second Joint Action on Antimicrobial Resistance and Healthcare-Associated Infections (EU-JAMRAI-2), uniting 128 partners from 30 countries, represents a coordinated EU/EEA effort to curb AMR through a One Health approach. Although nearly all EU/EEA countries have an AMR National Action Plan, our initial assessment revealed that implementation remains constrained by limited resources, weak intersectoral coordination and fragmented leadership. EU-JAMRAI-2 addresses these challenges by promoting harmonized surveillance, strengthening infection prevention and control, applying behaviorally tailored stewardship interventions, ensuring sustainable access to essential antibiotics, and raising awareness among priority target audiences. By analyzing policy gaps and operational barriers, this paper underscores the need for stronger accountability and political commitment to translate strategies into sustainable action. Strengthening AMR governance through a unified European approach is essential to achieve effective, lasting progress against this silent pandemic.

## Introduction

Antimicrobial resistance (AMR) poses a serious global health threat to countries of all economic levels and is projected to claim 1.91 million global lives in 2050.^1^^,^^2^ Its impact on public heath, healthcare systems, food security, and economic stability is profound and cannot be neglected. In Europe, recent data suggest that infections due to antimicrobial-resistant bacteria already account for more than 865,000 cases and 35,000 deaths annually in 2022, according to the European Centre for Disease Prevention and Control (ECDC).^3^ This burden is not only high but also increasing over time and across countries, and the prevalence of multi-drug resistant organisms (MDRO) differs between Member States.^4^^,^^5^ Among 30 Member States, over 569 million extra hospital days linked to AMR are projected by 2050.^6^ Overall, the stakes for public health, animal welfare, and ecological balance are increasing, while healthcare costs and productivity losses are estimated at €1.5 billion each year according to the OECD.^7^

In September 2024, world leaders launched a Political Declaration on AMR at the United Nations General Assembly (UNGA), emphasizing the need for implementing a One Health (OH) approach into countries' National Action Plans (NAPs).^8^ It reaffirmed the necessity of national ownership that considers local contexts, priorities and needs, and highlighted the importance of effective political leadership in tackling AMR. The OECD demonstrated that a OH approach is effective and cost-efficient. More specifically, investing USD 4 purchasing power parity (PPP) per person annually in a policy package could avert more than 17,000 deaths, save over USD 9.4 billion PPP in healthcare expenditures, and yield approximately USD 13.8 billion PPP in productivity gains across 34 OECD and EU/EEA countries.^6^

Following the UNGA declaration, the Quadripartite organization (WHO, Food and Agriculture Organization (FAO), World Organisation for Animal Health (WOAH) and United Nations Environment Programme (UNEP), recently started a consultative process to establish an Independent Panel on Evidence for Action against AMR aiming to better inform and guide policy making and interventions to prevent and mitigate its impact.

In EU/EAA countries, despite the existence of OH NAPs, significant variability has been evidenced in their implementation, including gaps in environmental integration, operational details, monitoring mechanisms and funding.^9^ Therefore, it requires Member States to work in synergy and exchange good practices while taking into account each country's specificities to update their current strategies. The European Directorate-General for Health & Food Safety (DG SANTE) through the EU4Health programme, the Health Emergency preparedness and Response Authority (HERA) via pandemic preparedness initiatives, and the Directorate-General for Research and Innovation (DG RTD) have launched programmes to help Member States integrate One Health into policies, and define European research and innovation priorities in line with the quadripartite's One Health priority research agenda for AMR (AMR-OHPRA).^10^

Many factors contribute to AMR development, including not only antibiotic consumption but also anthropological and socioeconomic influences,^11^^,^^12^ meaning AMR is not only a health issue but a highly complex governance challenge, requiring responses that extend beyond the clinical sphere. Therefore, although continuous efforts are needed to improve infection prevention and control (IPC) and antimicrobial stewardship (AMS), we need to consider a holistic approach encompassing sustainable access to effective antibiotics, diagnostics, and vaccines, as well as behavioral changes and awareness raising, while strengthening an integrated OH surveillance. Moreover, as travels also contribute to AMR dissemination,^13^^,^^14^ particularly in the context of globalisation, AMR is now included in the regulation on serious cross-border threats to health.^15^ Lastly, governance is also a key element for the success of policies to curb AMR. Indeed, a recent study showed a relationship between governance quality, antibiotic consumption and reduction of AMR,^16^ indicating effective governance is not merely supportive but a decisive factor in combating AMR.

## Europe is setting up to tackle AMR

To address these specific drivers of AMR, the second Joint Action on Antimicrobial Resistance and healthcare-Associated Infections (EU-JAMRAI 2), led by France (INSERM), has been launched in 2024 for four years (running till the end of 2027). EU-JAMRAI 2 is backed by a €50 million grant from the European Commission and gathers 128 partners from 30 countries, including the 27 EU Member States as well as Iceland, Norway, and Ukraine. The EU-JAMRAI 2 mission is, through joint and coordinated action across Europe, to promote interdisciplinary collaboration among countries, institutions, and sectors, to safeguard the effectiveness of antimicrobials and protect public health, with the ambitious goal of changing the global approach to antimicrobial resistance through a One Health perspective.

The EU-JAMRAI-2 project addresses five interrelated areas of action. It strengthens engagement and coordination through a policy group of national Liaison Officers, enabling the identification of needs and gaps while facilitating collaboration and the exchange of good practices between countries. It also promotes the wider uptake of state-of-the-art infection prevention and control (IPC) measures for both community-acquired and healthcare-associated infections, as well as antimicrobial stewardship (AMS) strategies in various settings, and provides tools to support the implementation of behaviorally informed stewardship interventions. EU-JAMRAI-2 further supports the development of an integrated One Health surveillance system for AMR in Europe, including a dedicated dashboard for the animal sector. In addition, participating countries are working to improve access to essential antibiotics and vaccines and to foster awareness and understanding of AMR among professionals and the public across Europe. Together, these pillars ensure that ongoing national and EU-level efforts are coherent and mutually reinforcing. Yet their success will depend on stronger, well-coordinated governance across sectors and among countries, alongside efforts to overcome the diverse challenges faced in implementing National Action Plans effectively.

## Gaps analysis in European AMR policies

In 2019, the WHO European Region showed that the burden of AMR was significantly associated with the degree of NAP implementation.^17^ To take into consideration EU/EEA specific contexts, the EU-JAMRAI 2 conducted a structured needs-assessment survey in autumn 2024, coordinated by its work package on Member State Engagement and NAP development. The survey was distributed via a network of national Liaison Officers to 30 EU/EEA countries, each providing one coordinated response integrating inputs from human, animal, and environmental health sectors where possible. Respondents were Officials from ministries of health, national public health institutes, and, in some cases, food and veterinary authorities. The questionnaire addressed the structure, status, and challenges of national AMR action plans, as well as policy priorities and good practices, following DG SANTE's Overview Report and the recent Council Recommendation on strengthening EU action against AMR.

Of the 28 out of 30 countries, only eight reported having a comprehensive One Health National Action Plan on AMR sufficiently in place at the time of the survey. The majority reported that not all sectors (human, animal, and environmental health) are yet adequately represented in their NAPs, with many planning improvements in the coming years. Although still under analysis, the preliminary results have shown that the main challenges faced by countries for the implementation of a OH approach relate to resource and governance issues. Financial constraints–including lack of financial resources, inadequate budget allocation, or challenges in aligning resources and/or long-term funding–were reported by more than half of respondents. This was followed by a lack of human resources (n = 10; 35.7%) and challenges related to the functioning of intersectoral coordination mechanisms (ICM) (n = 8; 28.6%). Lack of high-level political commitment was also frequently cited.

Conversely, concerning the ICM, twelve Member States (42.8%) have already established mechanisms or platforms for regular communication and joint decision-making, including task forces and/or steering committees. The survey also highlighted that the main sectors affected by policy gaps or differences in policy comprehensiveness are the environment (n = 13; 46.4%) and plant health (n = 10; 35.7%). Additionally, costed operational plans (detailed plans linking concrete activities, responsibilities, and funding to ensure that the NAP can be effectively implemented) are missing in eight countries and still under development in 16, often lacking clear timelines, roles, and budgets. Monitoring and evaluation frameworks are absent in ten countries, and when present, they sometimes lack standardized indicators and data collection, hindering progress tracking. Yet we see examples of newly implemented NAPs, like those of Denmark, Finland, and Iceland, taking a practical approach of concretely prioritizing actions annually, allocating funding and having clear timelines.

The findings underline the considerable differences between Member States in both their progress and challenges, while also revealing opportunities for improvement and collaboration. Many Member States also reported good practices or specific expertise they could share. For this purpose, the EU-JAMRAI 2 has launched a support programme that includes masterclasses and thematic working groups to foster knowledge sharing and best practice exchange. Through this ‘hub’, the EU-JAMRAI 2 fosters cooperation between Member States and facilitates mutual learning, while recognizing that one size does not fit all and respecting that the significant differences in systems and contexts across countries must incentivize tailored actions.

## Antimicrobial stewardship (AMS) and infection prevention and control (IPC)

Policy development alone is not enough: AMS and IPC are foundational, synergistic strategies to curb AMR. By preventing infections in the first place and optimizing antibiotic use when infections occur, robust IPC and AMS together directly reduce opportunities for resistant pathogens to emerge and spread. In healthcare settings, effective IPC measures such as rigorous hygiene protocols, can prevent up to 70% of healthcare-associated infections according to WHO estimates.^18^ At the same time, well-structured AMS programmes aim to ensure that antibiotics are prescribed only when truly necessary, and that the choice, dosage, and duration of treatment are appropriate. Evidence shows that such stewardship interventions can safely reduce unnecessary antibiotic use and improve prescribing practices for hospital inpatients.^19^

Despite their proven benefits, translating AMS and IPC policies into practice across all European health systems remains challenging. As previously mentioned, many countries and healthcare facilities lack the dedicated human resources, funding, and training required for these programmes, and the shortcomings were further exposed by the COVID-19 pandemic, even in advanced healthcare systems.^18^ Nevertheless, progress is being made and EU-wide efforts over the past decade have contributed to a 23% decrease in overall human antibiotic consumption from 2012 to 2021, indicating some success in stewardship.^7^ Also, aggregated sales (mg/PCU) declined by 53.0% over this period, i.e. from 161.2 mg/PCU in 2011 to 75.8 mg/PCU in 2022.^20^ However, the concurrent 34% rise in hospital use of last-resort antibiotics (e.g., carbapenems) highlights the need for further optimization of antibiotic use in acute care.^7^ Therefore, European authorities have accordingly called for intensifying IPC practices and establishing robust AMS programmes in every country as part of the One Health response to this “silent pandemic”.

In this context, EU-JAMRAI 2 is supporting Member States to adopt state-of-the-art IPC guidelines for both community and hospital settings and to roll out comprehensive stewardship strategies tailored to local needs. This includes developing behaviorally informed tools to help bridge the gap between guidelines and prescriber practice, fostering sustained improvements in antibiotic prescribing. Particular attention is being given to traditionally under-resourced areas such as long-term care facilities and paediatrics, through dedicated training and capacity-building initiatives, to ensure that AMS and IPC principles are embedded across all healthcare sectors. The initiative adopts a OH approach, extending IPC and AMS principles beyond human healthcare to animal health and environmental sectors. In animal health, the project supports the development of harmonized IPC/biosecurity protocols for livestock and companion animals, linking them to antimicrobial use reduction strategies. For the environmental sector, it maps interventions to mitigate AMR spread through pathways like wastewater and manure management. By reinforcing these core competencies, EU-JAMRAI 2 and related programmes aim to substantially strengthen Europe's defense against AMR on the frontline of patient care.

## Improving access to important antibiotics and vaccines

Access to effective antibiotics is known to prolong life, reduce disability and healthcare expenses, and enable access to medical innovations.^21^ In addition, sustainable access to effective antibiotics is a prerequisite for stewardship measures. To improve access to antibiotics and vaccines, the EU-JAMRAI 2 identified clinically important human and veterinary antibiotics and/or veterinary vaccines with a national vulnerable supply in 16 countries and established an interactive map of focus products for the project (https://eu-jamrai.eu/access/). We then identified barrier(s) hindering sustainable access to each focus product in each country and are now working to develop and implement a wide range of appropriate interventions targeting the needs of each country and product.

## Integrated one health surveillance of AMR

Surveillance is another cornerstone of the actions to combat AMR, enabling the timely detection of emerging resistance patterns, the monitoring of AMR trends, the comparisons across countries, and informing targeted interventions. The WHO's Tricycle project, focusing on Extended-spectrum beta-lactamase producing *Escherichia coli*, has pioneered integrated surveillance across the human, animal and the environment sectors.^22^, ^23^, ^24^ In Europe, the Joint Inter-Agency Antimicrobial Consumption and Resistance Analysis (JIACRA) reports analyze data from humans and food-producing animals from the five European surveillance networks to better understand emergence and concomitant trends of antimicrobial resistance across Europe. The recent EU council recommendations support the development of integrated systems for the surveillance of AMR (and antimicrobial consumption) encompassing human health, animal health, plant health, food, wastewater and the environment.^25^ In this context, EU-JAMRAI 2 drives an integrated OH AMR surveillance across Europe, promoting efforts to develop human, veterinary and environmental surveillance, complementing other EU initiatives. Besides the EARS-Net surveillance of AMR in human health led by the ECDC, the EU-JAMRAI 2 extends the EARS-Vet network on AMR surveillance in diseased animals,^26^^,^^27^ initiated in the first EU-JAMRAI 1 and including now 40 partners from 17 countries. The EU-JAMRAI 2, in collaboration with the EU-WISH joint Action,^28^ is also building the EARS-Env network for environmental surveillance, notably in wastewater.

## Education and awareness raising

Lastly, societal engagement requires scaling up, targeting the education of healthcare professionals, animal health professionals and public awareness. In many countries, AMR topics are still absent from university curricula, and different studies outlined the need to include more training and education in the field of appropriate antimicrobial prescribing. To increase awareness and improve education from the primary school to the University, the EU-JAMRAI 2 designed a broad portfolio of awareness-raising actions, combining creativity, scientific accuracy, and inclusivity to reach large and diverse audiences, and working in collaboration with other stakeholders, science museums networks^29^ and initiatives such as the AMR narrative initiative.^30^ Furthermore, the EU-JAMRAI, in its first edition, crafted an AMR symbol to unite efforts. The symbol has become widely recognized and a source of European pride and unity.

## Call for strengthening AMR one health governance in all countries

**Fig. 1:**
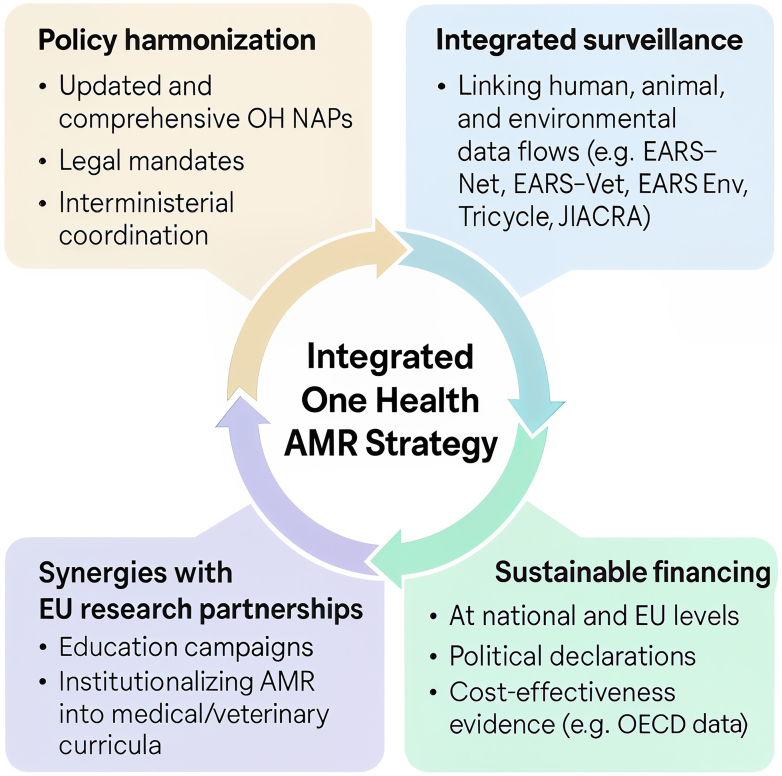
**EU-JAMRAI-2 integrated One Health strategy: four pillars of the urgent call for action against antimicrobial resistance**. Abbreviations: AMR: antimicrobial resistance; EARS-Net: European Antimicrobial Resistance Surveillance Network; EARS-Env: European Antimicrobial Resistance Surveillance Network for Environment; EARS-Vet: European Antimicrobial Resistance Surveillance Network in Veterinary Medicine; JIACRA: Joint Interagency Antimicrobial Consumption and Resistance Analysis; NAPs: National Action Plans; OECD: Organisation for Economic Co-operation and Development; OH: One Health.

In light of the increase in antibiotic-resistant infections in the EU/EEA, it is clear that more urgent action is needed to address AMR and strengthen its governance. The current situation, characterized by funding challenges, and a limited intersectoral collaboration, poses significant threats to public health. Additionally, political commitment may also waver in times of global instability. Changes in the international landscape, including shifts in funding priorities (e.g., the USA's withdrawal from international health and education investments) and ongoing crises such as wars and climate change, risk diverting attention and sustainable resources away from AMR. The integrated approach developed by EU-JAMRAI-2 is summarized in Fig. 1 and illustrates the four pillars needed to address the urgent call for action against AMR. By facilitating evidence-based OH national actions, adapted to local culture and structures, EU-JAMRAI-2 aims to support sustained and coordinated efforts, helping European countries to build a robust and unified defense against AMR, while underscoring the need for stronger governance to ensure lasting impact.

To succeed, we need to foster synergies. Therefore, the EU-JAMRAI 2 experts call on policymakers, healthcare professionals, and all stakeholders to join forces to prioritize AMR and implement effective, long-lasting solutions.

## Contributors

BD and MCP contributed to the conception and design of the work, literature search, data interpretation, drafting of the manuscript, preparation of the figure, and critical revision of the text. SN, CvL, CA, LAI, SL, LC, LP, NGH, MGR, GvdL, AAF, BK, YL, AC contributed to the interpretation of data, critical revision of the manuscript for important intellectual content, and approval of the final version. All authors had access to and verified the underlying data, approved the submitted version, and agreed to be accountable for all aspects of the work.

Members of the EU-JAMRAI-2 Study Group contributed to the coordination, data collection, and expert input across participating countries, and approved the final version of the manuscript.

## Declaration of interests

BD, AC, LAI, CvL, LC, LP, NGH, SL, YL, BK, GvdL, MCP, MG, and SN declare no competing interests. CA reports institutional grants from the World Health Organization (Regional Office for Europe), the Norwegian Research Council (MIA project), the Norwegian Agency for Development Cooperation (Norad), the Canadian Institutes of Health Research (NFRFE-2022-0412), and the European Economic Area grant for AMR collaboration with Romania, all paid to the Norwegian Institute of Public Health. She also reports receiving honoraria for a presentation to the European Parliament and travel support from Uppsala University and the Maltese Medicines Agency. AAF reports grants from the COSME programme (European Union)–As a member of the RaDAR project: This project has received funding from the COSME Programme of the European Union, Grant Agreement N° 101036228.

## Appendix A Supplementary data

Translated Abstract French
