# Supplementary material for: Strengthening antimicrobial resistance governance in Europe: a coordinated one health approach
Source: Lancet Reg Health Eur. 2025 Nov 18;61:101540. doi: 10.1016/j.lanepe.2025.101540 (PMC12767864; doi:10.1016/j.lanepe.2025.101540)
Supplement: Translated Abstract French [file mmc1.docx]

# *This translation in French was submitted by the authors and we reproduce it as supplied. It has not been peer reviewed. Our editorial processes have only been applied to the original abstract in English, which should serve as reference for this manuscript.*

# Résumé

La résistance aux antimicrobiens (RAM) provoque plus de 35 000 décès chaque année dans l’Union européenne et l’Espace économique européen (UE/EEE) et pourrait entraîner jusqu’à 1,9 million de décès par an dans le monde d’ici 2050. La deuxième action conjointe sur la résistance aux antimicrobiens et les infections associées aux soins (EU-JAMRAI-2), qui réunit 128 partenaires issus de 30 pays, constitue un effort coordonné de l’UE/EEE pour lutter contre la RAM selon une approche « Une seule santé ». Bien que la quasi-totalité des pays de l’UE/EEE disposent d’un plan d’action national, notre évaluation initiale montre que leur mise en œuvre reste freinée par des ressources limitées, une coordination intersectorielle insuffisante et un leadership fragmenté. L’EU-JAMRAI-2 s’emploie à relever ces défis en promouvant une surveillance harmonisée et intégrée, en renforçant la prévention et le contrôle des infections, en développant des interventions comportementales pour un bon usage des antibiotiques, en garantissant un accès durable aux antimicrobiens essentiels et en sensibilisant les acteurs clés. En analysant les lacunes politiques et opérationnelles, cet article souligne la nécessité d’une plus grande responsabilisation et d’un engagement politique renouvelé pour transformer les stratégies en actions durables. Le renforcement de la gouvernance européenne face à la RAM est indispensable afin d’atteindre des objectifs concrets et durables contre cette pandémie silencieuse.
